# Infant feeding practices and sleep at 1 year of age in the nationwide ELFE cohort

**DOI:** 10.1111/mcn.13072

**Published:** 2020-09-10

**Authors:** Sabine Messayke, Camille Davisse‐Paturet, Sophie Nicklaus, Marie‐Noëlle Dufourg, Marie‐Aline Charles, Blandine de Lauzon‐Guillain, Sabine Plancoulaine

**Affiliations:** ^1^ Université de Paris, CRESS, Inserm, INRAE Paris France; ^2^ Centre des Sciences du Goût et de l'Alimentation, AgroSup Dijon, CNRS, INRAE Université de Bourgogne Franche‐Comté Dijon France; ^3^ Unité mixte Inserm‐Ined‐EFS Elfe, INED Paris France

**Keywords:** birth cohort, epidemiology, infant feeding, sleep

## Abstract

Sleep problems reported by parents affect 20% to 30% of infants. Few studies focused on the longitudinal association between infant feeding practices and sleep, especially in France. Analyses were based on 8,696 infants from the French national birth cohort ELFE. Collection of feeding practices from birth to 10 months allowed for the identification of trajectories of use of baby cereals and thickened formula by group‐based trajectory modelling and calculation of duration of any breastfeeding (BF) and age at complementary feeding introduction (CFI) excluding baby cereals. Total sleep duration (TSD), night waking (NW) and sleep onset difficulties (SOD) were reported at age 1. Associations between feeding and sleep were tested by multinomial logistic regressions. BF duration ≥6 months was associated with parent‐reported frequent NW, SOD and TSD ≤ 12 h/24 h at age 1. For TSD and SOD, this association was no longer significant after accounting for parental sleep‐related practices. Early use of baby cereals (≤5 months) was associated with poor sleep. Early CFI (<4 months) was associated with shorter TSD and SOD but not NW. Early use of thickened formula (only <6 months) was related to poor sleep at age 1 (NW and SOD), whereas late (around 6 months) use of thickened formula was associated with better sleep. BF duration ≥6 months was related to poor sleep at age 1 but not after adjustment on 1‐year parental sleep‐related practices except for NW. The use of baby cereals or early CFI was not related to better sleep at age 1.

**Key messages**

- Breastfeeding duration ≥ 6 months was related to more sleep problems at age 1, but parental sleep‐related practices seemed to explain the link with sleep duration and sleep onset difficulties.
- Early baby‐cereals introduction and early complementary feeding introduction (<4 months) are not related to better sleep at age 1.
- Specific counselling on parental sleep‐related practices should be given to breastfeeding mothers.

## 1 INTRODUCTION

Sleep is a key domain of developmental processes of early life and is fundamental for cognitive and physical growth of infants and children (Cappuccio et al., 2008; Gruber et al., 2012; Reynaud, Vecchierini, Heude, Charles, & Plancoulaine, 2018). Frequent sleep onset difficulties, frequent night waking and short sleep duration as reported by parents and defining subjective sleep problems are prevalent for children less than 3 years old, with prevalence ranging from 20% to 30% (Bruni et al., 2014; Byars, Yolton, Rausch, Lanphear, & Beebe, 2012; Sette, Baumgartner, Ferri, & Bruni, 2017). Longitudinal studies showed that the presence of parent‐reported sleep problems (sleep onset difficulties, night waking and short sleep duration) in preschool and school‐aged children was positively associated with high scores on validated scales measuring emotional symptoms, hyperactivity/inattention (Cappuccio et al., 2008; Reynaud et al., 2018) and conduct problems later in childhood (Al Mamun et al., 2012; Byars et al., 2012; Meltzer, Plaufcan, Thomas, & Mindell, 2014).

Several studies reported associations between infant feeding and parent‐reported infant sleep, with different results according to infant age and study design. Hence, cross‐sectional studies reported that breastfeeding was associated with more frequent night waking among infants aged less than 6 months as compared with formula‐feeding (Figueiredo, Dias, Pinto, & Field, 2017; Galbally, Lewis, McEgan, Scalzo, & Islam, 2013; Huang et al., 2016; Hysing et al., 2014; Ramamurthy et al., 2012) but not with sleep duration (Figueiredo et al., 2017) or difficulties falling asleep (Galbally et al., 2013). However, longitudinal studies showed that the feeding method (breastfeeding versus formula feeding) at 6 months was associated neither with night waking nor with reported sleep duration between 18 and 24 months (Hysing et al., 2014; Nevarez, Rifas‐Shiman, Kleinman, Gillman, & Taveras, 2010). By comparing actigraphy data and maternal reports, a recent study showed that mothers who formula‐fed between age 1 and 4.5 months tended to overestimate their infants' night sleep duration as compared with mothers who breastfed (Rudzik, Robinson‐Smith, & Ball, 2018). More recently, predominant breastfeeding for more than 4 months was found associated with low risk of persistent reported sleep‐onset difficulties between age 2 and 5 years (Murcia et al., 2019).

The World Health Organization (WHO) recommends complementary feeding introduction (CFI) at 6 months of age and not before 4 months of age (World Health Organization, 2003). For healthy infants born at term and living in Europe, the European Society for Pediatric Gastroenterology, Hepatology, and Nutrition recommends CFI between 4 and 6 months for infants showing good neuromuscular capacity (Fewtrell et al., 2017).

However, mothers may introduce solid foods at an early stage with the objective of encouraging consumption of greater volumes during the day and letting their infants sleep more at night (Alder et al., 2004; Clayton, Li, Perrine, & Scanlon, 2013; Scott, Binns, Graham, & Oddy, 2009). A secondary analysis of children included in a population‐based randomized clinical trial on early solid food introduction, especially allergenic foods, conducted in England found that infants who were introduced to solid foods before age 4 months slept 7 min more on average at night in the intention‐to‐treat analysis (Perkin et al., 2018). However, only 42% of the children achieved the protocol in the early introduction group. The EDEN mother–child cohort also showed that CFI before age 4 months was related to low risk of short sleep duration between age 2 and 5 years (Murcia et al., 2019). However, other studies found the opposite, with introduction of solid food before 4 months being associated with short sleep duration at age 1 (Nevarez et al., 2010). Others showed that infants who were fed solid food during the day between 6 months and 1 year of age were less likely to be fed at night but not less likely to wake, with no difference in infant sleep observed between formula‐fed and breastfed infants (Brown & Harries, 2015). Besides the feeding methods or the introduction of solid foods, adding baby cereals into infants' bottles before bedtime among 1‐ to 4‐month‐old infants was not associated with their sleep (Macknin, Medendorp, & Maier, 1989; Murcia et al., 2019). The introduction of solids during the day showed no association with night waking frequency, but it was associated with low frequency of infant night feeding (Brown & Harries, 2015). In the literature, one study focused on thickened formula and infant sleep and reported no association with sleep developmental trajectories between age 2 and 5 years (Murcia et al., 2019).

Furthermore, several studies of the first year of life have shown that parents' sleep‐related behaviours, for example, parental presence when the child falls asleep, feeding before sleep, sucking a pacifier and sleeping in the parents' room, are positively associated with parent‐reported infant sleep problems (Hysing et al., 2014; Messayke et al., 2019; Mindell, Leichman, & Walters, 2017; Paul et al., 2017; Tikotzky, 2017). Sleeping in the parents' room is recommended for infants <6 months old to decrease the risk of sudden infant death syndrome (American Academy of Paediatrics, 2016). After this age, parents sharing the room with their infant reported more sleep problems than those whose infant slept in a separate room, but infant sleep characteristics on actigraphy did not differ by sleep location (Mindell et al., 2017). In France, about 15% of infants age 1 year share their parents' bedroom on a regular basis (Messayke et al., 2019).

In this context, the aim of the current study was to examine the association between feeding practices including well‐known practices (breastfeeding and complementary feeding) as well as less‐studied practices (use of baby cereals, use of thickened formula), focusing on their dynamics up to 10 months, and sleep quantity or quality of infants at age 1.

## 2 SUBJECTS AND METHODS

### 2.1 Population study

The study was based on data from the ‘Etude longitudinale française depuis l'enfance’ (ELFE) study, a nationwide, multidisciplinary, birth cohort study, that included 18,329 children born in a random sample of 349 maternity units in mainland France in 2011 (Charles et al., 2019). Inclusion criteria were singleton or twins born after 33 weeks' gestation to mothers aged 18 years or older.

Participating mothers provided written consent for their own and their child's participation. Fathers signed the consent for the child participation when present on inclusion days or were informed about their rights to oppose. The ELFE study received approval from the Consultative Committee for the Treatment of Information for Health Research (Comité Consultatif sur le Traitement des Informations pour la Recherche en santé), the National Data Protection Authority (Commission Nationale Informatique et Libertés) and the National Statistics Council.

### 2.2 Infant feeding

Infant feeding practices were collected at birth by face‐to‐face interview, at 2 months by phone interview and then monthly from 3 to 10 months by internet/paper questionnaires. Data on breastfeeding were updated by the 1‐ and 2‐year phone interviews.

From these data, any breastfeeding duration was calculated (Wagner et al., 2019) as was the age at CFI excluding baby cereals (Bournez et al., 2018). Any breastfeeding duration was considered a four‐category variable (never, <1, 1 to <3, 3 to <6 and ≥6 months) and the age at CFI excluding baby cereals was considered a three‐category variable (<4, 4 to 6 and >6 months) according to ESPGHAN and WHO recommendations (Fewtrell et al., 2017; World Health Organization, 2003).

Because some parents could add cereals to the infant's bottle or could use thickened formula to encourage sleep, we also focused on the use of baby cereals from 3 to 10 months (at least several times in the last month) and the use of thickened formula (from 2 to 10 months). The use of thickened formula was derived each month from the brand and the name of the infant formula used, when relevant (de Lauzon‐Guillain et al., 2018). If a baby received only breast milk at a given month, it was considered a nonuser of thickened formula at this month. Both variables were considered longitudinally as described in the statistical analysis section.

### 2.3 Sleep quantity and quality

Data on sleep quantity and quality were collected during the 1‐year follow‐up. Total sleep duration over 24 h was calculated based on the answers in hours and minutes to the following questions: ‘How much does your child sleep at night (on average)?’ and ‘How much does your child sleep in the day (on average). Add up all the naps taken in the day’. Total sleep duration was classified into three groups: ≤12 h/24 h, >12 to 14 h/24 h, and >14 h/24 h. Although the U.S. recommendations for healthy sleep duration are from 11 to 14 h for infants at age 1 (Paruthi et al., 2016), to define short sleep duration, we used the threshold of ≤12 h/24 h, recommended by the National Sleep Foundation at the time of the study (Bruni & B. P., 2017; Mindell, Meltzer, Carskadon, & Chervin, 2009). This less restrictive cut‐off allowed us to consider a larger sample size for this category (14.7% vs. 5.4% for <11 h/24 h at age 1), even if it is probably less specific. Night waking frequency was assessed by the question ‘Does your child have periods when he/she wakes up at night?’ If yes ‘On this week, how many nights has your baby woken up (if the child was ill this week, ask about a week when the child wasn't ill)?’ Possible answers were ‘never’, ‘1 or 2 nights’, ‘3 to 6 nights’, and ‘every night’. The last two categories were grouped into ‘>2 nights/week’ (Zuckerman, Stevenson, & Bailey, 1987). The night‐time schedule was not defined in the questionnaire, and answers were based on the parents' understanding and reflect their night waking perception. This question was asked just after the ones on sleep bedtime and wake time, thereby suggesting that it was about waking occurring during nocturnal sleep. Sleep onset difficulties were assessed by the question ‘When you put your child to bed, does he/she have problems going to sleep (for example, they call you or cry for a long time)?’ Possible answers were ‘never’, ‘sometimes’ and ‘always’. As for night waking, the answers were based on parents' perception.

Information on sleep‐related variables was also collected at age 1. The variables were previously described elsewhere (Messayke et al., 2019). Briefly, sleep‐related variables were the use of a pacifier or finger sucking to sleep (coded as none, thumb/finger only, pacifier only, both pacifier and thumb/finger) and the parental sleep‐related practices sleep arrangement (i.e., where the infant falls asleep and ends the sleep [falls asleep and ends sleep in own bed/crib, falls asleep in their own bed/crib but ends it in the parent's bed, falls asleep and ends sleep elsewhere than in their own bed/crib], sleeping in the parent's room (no, yes) and parental presence when falling asleep (no, yes). Only night waking frequency was available in the questionnaire at 2 months, coded as every night, one night over two, sometimes and never, and then classified into three groups (never, sometimes and often including one night over two and every night).

### 2.4 Infant and parental characteristics

Because family data were more completely collected during the 2‐month interview than during the maternity interview and family sociodemographic characteristics only marginally evolved within 2 months, we considered data collected 2 months postpartum in our analyses. The sociodemographic characteristics collected during the maternity stay were used only in case of missing values at the 2‐month follow‐up.

Maternal characteristics were as follows: country of birth (born in France or abroad), BMI before pregnancy (<18.5, 18.5–24.9, 25.0–29.9, ≥30 kg/m^2^), psychological difficulties during pregnancy (no, yes), smoking status (never smoker, smoker only before pregnancy, smoker only in early pregnancy, smoker even in late pregnancy), primiparous (no, yes), age at delivery (<25, 25–29, 30–34, >34 years), education attainment (according to the International Standard Classification of Education: below secondary school, upper secondary school, intermediate, higher), working status (not working during pregnancy, not yet returned to work at 2 months, returned to work at 2 months) and single motherhood (no, yes). The family income per consumption unit (CU) at 2 months was classified as ≤ €750, €751–1,111, €1,112–1,500, €1,501–1,944, €1,945–2,500, > €2,500 per month.

Infant characteristics were collected from the medical records: sex, gestational age and birth weight. The existence of gastrointestinal reflux (no, yes) was reported by parents at the 2‐month follow‐up.

### 2.5 Sample selection

Among the 18,329 infants present in the study at birth, infants whose parents withdrew consent within the first year (*n* = 57) or for whom the eligibility criteria were not verified (*n* = 478) were excluded, resulting in 17,794 eligible infants. We excluded multiple pregnancies (*n* = 548), and infants born preterm (*n* = 756), leading to a sample of 16,490 infants. We then excluded infants with no information (*n* = 5,192) on complementary feeding questionnaires, leading to a sample of 11,298 infants.

We computed separately two sets of trajectories for feeding practices based on the maximum available information for each one. Thus, we excluded 1,091 infants from the 11,298 infants to compute the trajectories for the use of thickened formula between 2 and 10 months and 989 from the 11,298 infants to compute the trajectories for the use of baby cereals between 3 and 10 months. We then retained for further steps infants without missing data on both sets of trajectories, leading to a sample of 10,108 infants.

We excluded families who did not reply at the 1‐year follow‐up (*n* = 531), those with missing data on at least one sleep quantity/quality variable at age 1 (i.e., sleep duration, night waking and sleep onset difficulties; *n* = 242) and those with missing data on infant feeding (i.e., breastfeeding duration; *n* = 84), and age at CFI excluding baby cereals (*n* = 109), leading to a sample of 9,142 infants. The complete‐case sample, excluding infants with missing data for potential confounders (*n* = 446) was 8,696 infants (Figure S1).

### 2.6 Statistical analysis

All statistical analyses were performed with SAS 9.3 (SAS, Cary, NC, on AIX 7.1 platform). The significance level was set at *p* ≤ 0.05. The comparison of included and excluded participants and the bivariate analyses between infant feeding and sleep quantity/quality involved chi‐square test and Student *t* test.

#### 2.6.1 Group based trajectory modelling

The group‐based trajectory method (PROC TRAJ procedure) (Nagin, 2005) was used to describe longitudinal infant feeding practices over the time. This data‐driven method aims at identifying within the studied population, using maximum likelihood and based on each series of responses, groups that evolved according to different patterns. Polynomial equations, defining the trajectories, were used to model the relationship between age and infant feeding practices. Different models were then computed and compared by using the Bayesian Information Criteria (BIC) and favouring parsimony. The group‐based trajectory method allows for including missing data. Infants were included in the trajectory's elaboration if their parents had answered the questions regarding infant feeding practice for at least five time points in nine for the thickened formula milk and at least four time points in eight for the use of baby cereals.

**FIGURE 1:**
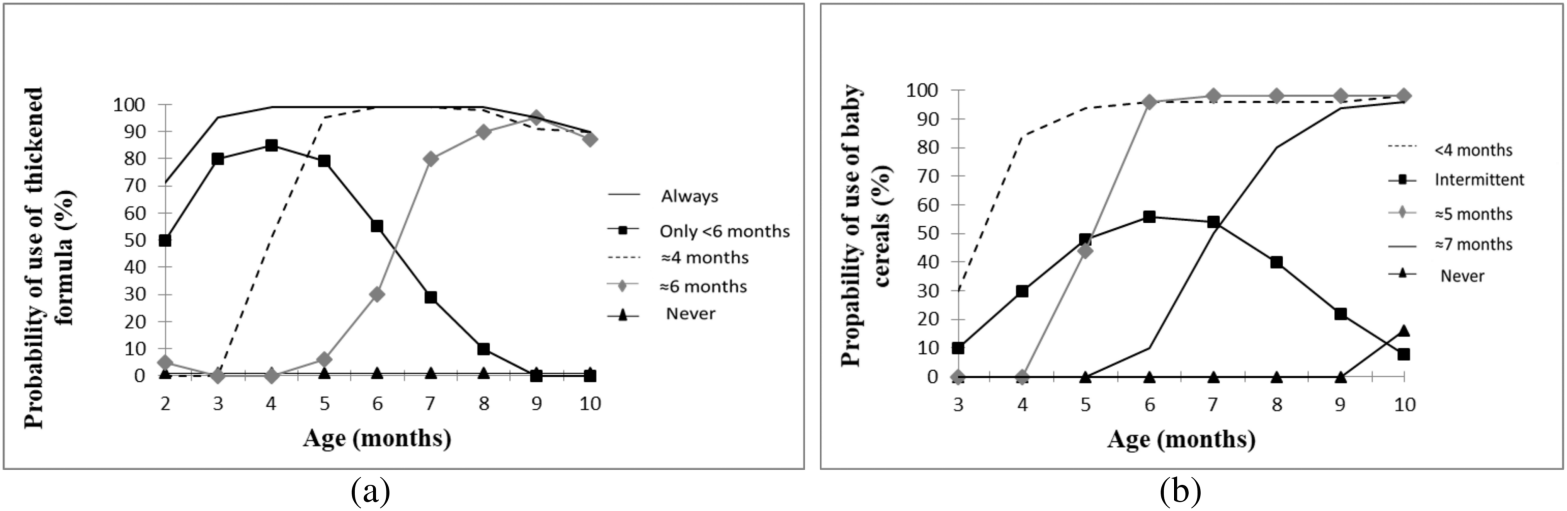
(a) Trajectories of use of thickened formula between 2 and 10 months in the ELFE nationwide cohort (*n* = 10,207). The ‘never’ use of thickened formula trajectory (55.4%) is depicted in black with triangles, the use of thickened formula ‘only before 6 months’ trajectory (7.7%) is depicted in black with squares, the ‘introduction around 4 months and persistence’ of thickened formula use trajectory (5.0%) is depicted in dashed line, the ‘introduction around 6 months and persistence’ of thickened formula use trajectory (12.1%) is depicted in grey with diamonds, and the ‘always’ use of thickened formula trajectory (19.8%) is depicted in full line. (b) Trajectories of use of baby cereals between 3 and 10 months in the ELFE nationwide cohort study (*n* = 10,309). The ‘never’ use of baby cereals trajectory (34.9%) is depicted in black with triangles, the ‘intermittent use’ of baby cereals trajectories (4.1%) is depicted in black with squares, the ‘introduction before 4 months and persistence’ of baby cereals use trajectory (11.1%) is depicted in dashed line, and the ‘introduction around 5 months and persistence’ of baby cereals use trajectory (21.3%) is depicted in grey with diamonds, the ‘introduction around 7 months and persistence’ of baby cereals use trajectory (28.4%) is depicted in full line

Concerning trajectory modelling of the use of thickened formula from age 2 to 10 months, the optimal model was a five‐group model (Figure 1a). The first trajectory labelled ‘never’ had a linear shape and represented 55.4% of the infants; the second, third, fourth and fifth trajectories had a cubic shape and were labelled ‘only before 6 months’, ‘introduction around 4 months and persistence’, ‘introduction around 6 months and persistence’ and ‘always’ trajectories and represented 7.7%, 5.0%, 12.1% and 19.8% of the infants, respectively.

The optimal trajectory modelling of the use of baby cereals from age 3 to 10 months was also a five‐group model (Figure 1b). The first trajectory labelled ‘never’ had a cubic shape and represented 34.9% of the infants; the second trajectory labelled ‘intermittent use’ had a quadratic shape and represented 4.1% of the infants; the third, fourth and fifth trajectories had a cubic shape and were labelled ‘introduction before 4 months and persistence’, ‘introduction around 5 months and persistence’ and ‘introduction around 7 months and persistence’ trajectories and represented 11.1%, 21.3% and 28.4% of infants, respectively. Nagin's recommended criteria for goodness of fit were met for all groups (Table S1).

#### 2.6.2 Multivariate analyses

The associations between infant feeding practices (breastfeeding duration, trajectories of use of baby cereals between age 3 and 10 months, age of CFI excluding baby cereals, and trajectories of use of thickened formula between age 2 and 10 months) and sleep quantity/quality (sleep duration, frequent sleep onset difficulties and frequent night waking) were analysed by multinomial multivariate logistic regression models. Potential confounding factors included in the models were identified from the literature. A Directed Acyclic Graph (DAG) was drawn showing the known relationships between breastfeeding, CFI and sleep characteristics. We added our new hypothesis regarding the use of baby cereals and thickened formula. The minimum set of adjustment variables was selected by using the DAG method (Ferguson et al., 2020). All models were thus adjusted for maternal characteristics (country of birth, BMI before pregnancy, psychological difficulties during pregnancy, smoking status during pregnancy, primiparous, age at delivery, education attainment, working status at 2 months, single motherhood), family income, infant characteristics (sex, birth weight and gastrointestinal reflux at 2 months) and variables related to study design (maternal region of residence, size of maternity unit and wave of recruitment).

Analyses were performed separately for each sleep quantity/quality parameter (sleep duration, night waking and sleep onset difficulties). First, we analysed the association between all infant feeding practices considered simultaneously with each infant sleep quality/quantity parameter, adjusted for the potential confounding factors presented above and selected using the DAG method (Model 1). Second, we additionally adjusted for infant night waking frequency at 2 months (Model 2) to account for the infant sleep quality at an early age and address potential reverse causation bias. Third, we additionally adjusted the models for the use of a pacifier or finger sucking at age 1 (Model 3) to account for infant soothing behaviours. Fourth, we additionally adjusted the models for the other infant sleep‐related variables at age 1 (i.e., infant sleep arrangement, sleeping in the parent's room and parental presence; Model 4), to adjust for parental sleep‐related practices. Because parental presence when the child falls asleep may be related to breastfeeding, interactions between breastfeeding duration and parental presence when the child falls asleep or need for a pacifier or thumb/finger sucking to fall asleep were tested for sleep quantity and quality.

To deal with missing data for potential confounding factors, we performed multiple imputations as a sensitivity analysis with the SAS software. This method assigned data to missing measurements based on the measurement of infants with similar profiles. We assumed that data were missing at random and generated five independent datasets with the fully conditional specification method (MI procedure, FCS statement, NIMPUTE option), then calculated pooled effect estimates (MIANALYSE procedure). These sensitivity analyses allowed us to assess the association between infant feeding and sleep quantity/quality on a sample of 9,142 infants at age 1.

### 2.7 Ethical considerations

The ELFE birth‐cohort was approved by the Consultative Committee for the Treatment of Information for Health Research (Comité Consultatif sur le Traitement des Informations pour la Recherche en santé), the National Data Protection Authority (Commission Nationale Informatique et Libertés) and the National Statistics Council.

## 3 RESULTS

### 3.1 Characteristics of the studied population

The studied population was restricted to those with complete information. As compared with excluded infants (*n* = 7,794), included infants (*n* = 8,696) were more frequently born to a mother with high education attainment (46% vs. 24%, *p* < 10^−4^), older age at delivery (31 vs. 30 years, *p* < 10^−4^), high family income (€1,814 vs. €1,437 per CU, *p* < 10^−4^), low BMI before pregnancy (23.3 vs. 23.7 kg/m^2^, *p* < 10^−4^) and primiparous (46% vs. 45%, *p* < 10^−4^). The two groups did not differ in child sex (*p* = 0.57). Table 1 provides maternal and infant characteristics for the included sample.

**TABLE 1:** Parental and child characteristics (*N* = 8,696)

	% (*N*) or mean (*SD*)
Maternal country of birth (abroad)	6.8% (588)
Single motherhood (yes)	2.0% (171)
Primiparous mother (yes)	46.4% (4,031)
Maternal age at delivery, years	
<25	7.8% (674)
25–29	24.8% (2,153)
30–34	41.6% (3,629)
>34	25.8% (2,240)
Maternal education attainment	
Below secondary	3.1% (275)
Upper secondary	26.2% (2,275)
Intermediate	25.0% (2,172)
Higher	45.7% (3,974)
Maternal working status	
Not working during pregnancy	3.7% (326)
Not yet returned to work at 2 months	91.0% (7,917)
Returned to work at 2 months	5.3% (453)
Familial income per consumption unit, per month	
≤€750	4.2% (362)
€751–1,111	10.2% (892)
€1,112–1,500	27.3% (2,372)
€1,501–1,944	27.5% (2,388)
€1,945–2,500	19.2% (1,673)
>€2,500	11.6% (1,009)
Maternal BMI before pregnancy, kg/m^2^	
<18.5	6.8% (588)
18.5–24.9	67.7% (5,897)
25–29.9	16.9% (1,466)
≥30	8.6% (745)
Maternal psychological difficulties during pregnancy (yes)	11.3% (983)
Maternal smoking status	
Never smoker	58.3% (5,076)
Only before pregnancy	26.4% (2,295)
Only in early pregnancy	3.6% (310)
Even in late pregnancy	11.7% (1,015)
Sex (girl)	49.0% (4,259)
Gestational age (weeks)	39.5 (1.1)
Birth weight (kg)	3.388 (0.444)
Gastrointestinal reflux reported at 2 months (yes)	20.1% (1,747)

### 3.2 Associations between feeding practices and sleep quantity/quality

Frequencies of each feeding practice and infant sleep quantity/quality are in Table S2.

#### 3.2.1 Total sleep duration

On multivariate analyses (Model 1, Table 2), breastfeeding duration ≥6 months, early use of baby cereals (before 4 months or around 5 months and persistence) and early age at CFI (<4 months) were associated with increased odds of parent‐reported sleep duration of ≤12 h/24 h compared with intermediate duration (>12 to 14 h/24 h). Conversely, breastfeeding duration ≥6 months and early age at CFI (<4 months) were associated with reduced odds of parent‐reported sleep duration of >14 h/24 h. No association was observed between the use of thickened formula and total sleep duration.

**TABLE 2:** Multivariate associations between feeding practices and total sleep duration at 1 year old (*N* = 8,696; reference: >12 to 14 h/24 h)

	Model 1			Model 2^a^			Model 3^b^			Model 4^c^		
	≤12 h	>14 h		≤12 h	>14 h		≤12 h	>14 h		≤12 h	>14 h	
Breastfeeding duration			<10^−5^			10^−3^			0.05			0.65
Never	0.9 [0.7–1.1]	1.0 [0.8–1.1]		1.0 [0.8–1.2]	0.9 [0.8–1.1]		1.0 [0.8–1.2]	0.9 [0.8–1.1]		1.0 [0.8–1.2]	0.9 [0.8–1.1]	
<1 mo	1.0 [0.8–1.3]	1.0 [0.9–1.2]		1.1 [0.9–1.4]	1.0 [0.8–1.2]		1.1 [0.9–1.4]	1.0 [0.8–1.2]		1.0 [0.8–1.3]	1.0 [0.8–1.2]	
1 to <3 mo	1 [ref]	1 [ref]		1 [ref]	1 [ref]		1 [ref]	1 [ref]		1 [ref]	1 [ref]	
3 to <6 mo	1.0 [0.8–1.2]	1.0 [0.8–1.2]		1.0 [0.8–1.2]	1.0 [0.9–1.2]		1.0 [0.8–1.2]	1.0 [0.9–1.2]		0.9 [0.7–1.1]	1.0 [0.9–1.2]	
≥6 mo	1.3 [1.0–1.6]	0.8 [0.7–0.9]		1.2 [1.0–1.5]	0.8 [0.7–1.0]		1.2 [0.9–1.4]	0.9 [0.7–1.0]		0.9 [0.7–1.1]	0.9 [0.8–1.1]	
Trajectories of use of baby cereals between 3 and 10 mo			<10^−3^			10^−2^			10^−2^			0.02
Never	1 [ref]	1 [ref]		1 [ref]	1 [ref]		1 [ref]	1 [ref]		1 [ref]	1 [ref]	
Intermittent use	1.2 [0.8–1.7]	0.9 [0.7–1.2]		1.2 [0.8–1.7]	0.9 [0.7–1.2]		1.2 [0.8–1.7]	0.9 [0.7–1.2]		1.2 [0.8–1.7]	0.9 [0.7–1.2]	
Intro. <4 mo and perst.	1.6 [1.3–2.0]	0.9 [0.8–1.1]		1.6 [1.2–2.0]	0.9 [0.8–1.1]		1.6 [1.2–2.0]	1.0 [0.8–1.2]		1.6 [1.2–2.0]	1.0 [0.8–1.2]	
Intro. ≈5 mo and perst.	1.2 [1.0–1.4]	0.9 [0.8–1.1]		1.2 [1.0–1.4]	0.9 [0.8–1.1]		1.2 [1.0–1.4]	1.0 [0.8–1.1]		1.2 [1.0–1.5]	0.9 [0.8–1.1]	
Intro. ≈7 mo and perst.	1.0 [0.9–1.2]	1.0 [0.9–1.1]		1.1 [0.9–1.2]	1.0 [0.9–1.1]		1.1 [0.9–1.2]	1.0 [0.9–1.1]		1.1 [0.9–1.3]	1.0 [0.8–1.1]	
Complementary feeding introduction excluding baby cereals			0.01			0.01			0.02			0.05
<4 mo	1.3 [1.0–1.6]	0.8 [0.6–1.0]		1.2 [1.0–1.6]	0.8 [0.6–1.0]		1.2 [1.0–1.6]	0.8 [0.6–1.0]		1.2 [0.9–1.5]	0.8 [0.6–1.0]	
4–6 mo	1 [ref]	1 [ref]		1 [ref]	1 [ref]		1 [ref]	1 [ref]		1 [ref]	1 [ref]	
>6 mo	1.1 [0.9–1.3]	1.1 [1.0–1.2]		1.1 [0.9–1.3]	1.1 [1.0–1.3]		1.1 [0.9–1.3]	1.1 [1.0–1.3]		1.1 [0.9–1.3]	1.1 [1.0–1.3]	
Trajectories of use of thickened formula between 2 and 10 mo			0.16			0.23			0.21			0.17
Never	1 [ref]	1 [ref]		1 [ref]	1 [ref]		1 [ref]	1 [ref]		1 [ref]	1 [ref]	
Only <6 mo	1.1 [0.9–1.5]	0.8 [0.6–0.9]		1.1 [0.9–1.5]	0.8 [0.6–1.0]		1.1 [0.9–1.5]	0.8 [0.6–1.0]		1.2 [0.9–1.5]	0.8 [0.6–1.0]	
Intro. ≈4 mo and perst.	1.0 [0.7–1.3]	0.9 [0.7–1.1]		1.0 [0.7–1.3]	0.9 [0.7–1.1]		1.0 [0.7–1.3]	0.9 [0.7–1.1]		1.1 [0.8–1.4]	0.9 [0.7–1.1]	
Intro. ≈6 mo and perst.	0.9 [0.8–1.2]	0.9 [0.8–1.0]		0.9 [0.8–1.2]	0.9 [0.8–1.0]		0.9 [0.8–1.2]	0.9 [0.8–1.0]		1.0 [0.8–1.2]	0.9 [0.8–1.0]	
Always	1.0 [0.9–1.3]	0.9 [0.8–1.1]		1.0 [0.8–1.2]	1.0 [0.8–1.1]		1.0 [0.8–1.3]	0.9 [0.8–1.1]		1.8 [0.9–1.3]	0.9 [0.8–1.1]	

]*Note.* Data are adjusted odds ratios (aORs) [95% confidence intervals (CIs). The multinomial multiple logistic regression (Model 1) was adjusted for maternal characteristics (country of birth, BMI before pregnancy, psychological difficulties during pregnancy, smoking status during pregnancy, primiparous, age at delivery, education attainment, working status, single), familial income, and infant characteristics (sex, birth weight, and gastro‐intestinal reflux) and variables related to study design (maternal region of residence, size of maternity unit and wave of recruitment). Significant association (*p* < .05).

Abbreviations: mo, month; intro., introduction; perst., persistence.

^a^ Model 2: Model 1also adjusted on night waking at 2 months.

^b^ Model 3: Model 2 also adjusted on the use of sucking the pacifier/thumb at 1 year.

^c^ Model 4: Model 3 also adjusted on the infant sleep‐related variables (sleep arrangement, sleeping in parent's room and parental presence).

After adjustment for night waking at 2 months (Model 2), associations remained similar. After additional adjustment on the use of pacifier or sucking the thumb to fall asleep (Model 3), the association between breastfeeding and sleep durations remained stable but became less significant and was no longer significant after the introduction of parental sleep‐related practices (parental presence when the child falls asleep, sleeping in the parent's room and sleep arrangement) in the last model (Model 4). The other associations remained similar but became borderline significant for age at CFI.

#### 3.2.2 Night waking

Breastfeeding duration ≥6 months and an early use of baby cereals (intermittent use and introduction before 4 months and persistence) were associated with increased odds of frequent night waking (>2 nights/week) as compared with infants who never experienced night waking (Model 1, Table 3). Age at CFI was not associated with odds of frequent night waking. An early use of thickened formula (only before 6 months) was associated with increased odds of few nights waking and late introduction of thickened formula (introduction around 6 months and persistence) was associated with reduced odds of frequent night waking.

**TABLE 3:** Multivariate associations between feeding practices and night waking at 1 year old (*N* = 8,696; reference: never)

	Model 1			Model 2^a^			Model 3^b^			Model 4^c^		
	1–2 nights/week	>2 nights/week		1–2 nights/week	>2 nights/week		1–2 nights/week	>2 nights/week		1–2 nights/week	>2 nights/week	
Breastfeeding duration			<10^−4^			<10^−4^			<10^−4^			<10^−4^
Never	0.9 [0.7–1.0]	0.8 [0.7–1.0]		0.9 [0.8–1.1]	1.0 [0.8–1.2]		0.9 [0.8–1.1]	1.0 [0.8–1.2]		0.9 [0.8–1.0]	0.9 [0.8–1.2]	
<1 mo	0.8 [0.7–1.0]	0.9 [0.7–1.1]		0.9 [0.7–1.0]	1.0 [0.8–1.2]		0.8 [0.7–1.0]	1.0 [0.8–1.2]		0.8 [0.7–1.0]	0.9 [0.7–1.1]	
1 to <3 mo	1 [ref]	1 [ref]		1 [ref]	1 [ref]		1 [ref]	1 [ref]		1 [ref]	1 [ref]	
3 to <6 mo	0.9 [0.8–1.1]	1.2 [0.9–1.4]		0.9 [0.7–1.0]	1.1 [0.9–1.3]		0.9 [0.8–1.1]	1.1 [0.9–1.4]		0.9 [0.7–1.0]	1.0 [0.8–1.3]	
≥ 6 mo	1.1 [0.9–1.3]	2.1 [1.7–2.5]		1.0 [0.9–1.2]	1.8 [1.5–2.2]		1.1 [1.0–1.3]	1.9 [1.6–2.4]		1.1 [0.9–1.2]	1.4 [1.2–1.8]	
Trajectories of use of baby cereals between 3 and 10 mo			<10^−5^			<10^−4^			<10^−4^			<10^−3^
Never	1 [ref]	1 [ref]		1 [ref]	1 [ref]		1 [ref]	1 [ref]		1 [ref]	1 [ref]	
Intermittent use	1.2 [0.9–1.6]	1.5 [1.1–2.1]		1.2 [0.9–1.5]	1.4 [1.0–2.0]		1.1 [0.8–1.5]	1.3 [1.0–1.9]		1.1 [0.8–1.5]	1.4 [1.0–2.0]	
Intro. <4 mo and perst.	1.1 [0.9–1.3]	1.6 [1.3–2.0]		1.1 [0.9–1.3]	1.5 [1.2–1.9]		1.0 [0.9–1.3]	1.5 [1.2–1.9]		1.0 [0.8–1.3]	1.5 [1.2–1.9]	
Intro. ≈5 mo and perst.	1.1 [1.0–1.3]	1.2 [1.0–1.4]		1.1 [1.0–1.3]	1.1 [1.0–1.3]		1.1 [1.0–1.3]	1.1 [1.0–1.3]		1.1 [1.0–1.3]	1.1 [0.9–1.3]	
Intro. ≈7 mo and perst.	1.1 [0.9–1.2]	0.9 [0.8–1.1]		1.1 [0.9–1.2]	0.9 [0.8–1.1]		1.0 [0.9–1.2]	0.9 [0.8–1.1]		1.1 [0.9–1.2]	1.0 [0.8–1.1]	
Complementary feeding introduction excluding baby cereals			0.19			0.21			0.24			0.25
<4 mo	1.3 [1.0–1.6]	1.2 [0.9–1.5]		1.3 [1.0–1.6]	1.1 [0.9–1.5]		1.3 [1.0–1.6]	1.1 [0.9–1.5]		1.2 [1.0–1.6]	1.0 [0.8–1.4]	
4–6 mo	1 [ref]	1 [ref]		1 [ref]	1 [ref]		1 [ref]	1 [ref]		1 [ref]	1 [ref]	
>6 mo	0.9 [0.8–1.1]	1.0 [0.8–1.1]		0.9 [0.8–1.1]	1.0 [0.8–1.1]		0.9 [0.8–1.1]	1.0 [0.8–1.1]		0.9 [0.8–1.1]	1.0 [0.8–1.1]	
Trajectories of use of thickened formula between 2 and 10 mo			<10^−3^			<10^−3^			<10^−3^			<10^−3^
Never	1 [ref]	1 [ref]		1 [ref]	1 [ref]		1 [ref]	1 [ref]		1 [ref]	1 [ref]	
Only <6 mo	1.4 [1.1–1.7]	1.1 [0.8–1.4]		1.4 [1.1–1.6]	1.0 [0.8–1.3]		1.3 [1.1–1.6]	1.0 [0.8–1.3]		1.3 [1.1–1.6]	1.0 [0.8–1.3]	
Intro. ≈4 mo and perst.	1.0 [0.8–1.3]	1.1 [0.9–1.5]		1.0 [0.8–1.2]	1.1 [0.8–1.4]		1.0 [0.8–1.2]	1.1 [0.8–1.4]		1.0 [0.8–1.3]	1.2 [0.9–1.6]	
Intro. ≈6 mo and perst.	1.1 [1.0–1.3]	0.8 [0.6–0.9]		1.1 [1.0–1.3]	0.8 [0.6–0.9]		1.1 [0.9–1.3]	0.8 [0.6–0.9]		1.1 [0.9–1.3]	0.8 [0.6–0.9]	
Always	1.0 [0.8–1.1]	0.9 [0.8–1.1]		1.0 [0.8–1.1]	0.9 [0.7–1.1]		0.9 [0.8–1.1]	0.9 [0.7–1.1]		1.0 [0.8–1.1]	0.9 [0.8–1.1]	

*Note.* Data are adjusted odds ratios (aORs) [95% confidence intervals (CIs). The multinomial multiple logistic regression (Model 1) was adjusted for maternal characteristics (country of birth, BMI before pregnancy, psychological difficulties during pregnancy, smoking status, primiparous, age at delivery, education attainment, working status, single), familial income, and infant characteristics (sex, birth weight, and gastro‐intestinal reflux) and variables related to study design (maternal region of residence, size of maternity unit and wave of recruitment). Significant association (*p* < .05).

Abbreviations: mo, month; intro., introduction; perst., persistence.

^a^ Model 2: Model 1also adjusted on night waking at 2 months.

^b^ Model 3: Model 2 also adjusted on the use of sucking the pacifier/thumb at 1 year.

^c^ Model 4: Model 3 also adjusted on the infant sleep‐related variables (sleep arrangement, sleeping in parent's room and parental presence).

After adjustment on night waking at 2 months (Model 2), the use of a pacifier or sucking the thumb to fall asleep (Model 3) and infant sleep‐related variables (Model 4), the association between breastfeeding duration ≥6 months and frequent night waking weakened, especially in the last model, but remained significant. The other associations did not change.

#### 3.2.3 Sleep onset difficulties

Breastfeeding duration ≥6 months was associated with increased odds of both few (sometimes) and frequent (always) sleep onset difficulties, but never being breastfed was associated with reduced odds of frequent sleep onset difficulty as compared with infants who never experienced sleep onset difficulties (Model 1, Table 4). An early use of baby cereals (<4 months or around 5 months and persistent) was associated with increased odds of frequent sleep onset difficulties, whereas the introduction of baby cereals around 7 months was associated with increased odds of both few and frequent sleep onset difficulties. An early age at CFI (<4 months) was associated with increased odds of frequent sleep onset difficulties and a late age at CFI (>6 months) was associated with reduced odds of sleep onset difficulties. An early use of thickened formula (only <6 months) was associated with increased odds of sleep onset difficulties, but the introduction around 6 months was associated with reduced odds of sleep onset difficulties.

**TABLE 4:** Multivariate associations between feeding practices and sleep onset difficulties at 1 year old (*N* = 8,696; reference: never)

	Model 1			Model 2^a^			Model 3^b^			Model 4^c^		
	Sometimes	Always		Sometimes	Always		Sometimes	Always		Sometimes	Always	
Breastfeeding duration			<10^−4^			<10^−4^			<10^−4^			0.17
Never	0.9 [0.8–1.1]	0.7 [0.6–0.9]		1.0 [0.8–1.1]	0.8 [0.6–1.0]		1.0 [0.8–1.1]	0.8 [0.6–1.0]		0.9 [0.8–1.1]	0.8 [0.6–1.0]	
<1 mo	1.1 [0.9–1.2]	1.0 [0.7–1.2]		1.1 [0.9–1.3]	1.0 [0.8–1.3]		1.1 [0.9–1.3]	1.0 [0.8–1.3]		1.1 [0.9–1.3]	1.0 [0.7–1.3]	
1 to <3 mo	1 [ref]	1 [ref]		1 [ref]	1 [ref]		1 [ref]	1 [ref]		1 [ref]	1 [ref]	
3 to <6 mo	1.1 [0.9–1.2]	1.2 [0.9–1.5]		1.0 [0.9–1.2]	1.1 [0.9–1.4]		1.1 [0.9–1.2]	1.1 [0.9–1.4]		1.0 [0.9–1.2]	1.0 [0.8–1.3]	
≥6 mo	1.2 [1.0–1.4]	2.2 [1.8–2.8]		1.1 [1.0–1.3]	2.0 [1.6–2.6]		1.2 [1.0–1.4]	2.0 [1.5–2.5]		1.1 [0.9–1.3]	1.2 [0.9–1.6]	
Trajectories of use of baby cereals between 3 and 10 mo			0.03			0.06			0.07			0.01
Never	1 [ref]	1 [ref]		1 [ref]	1 [ref]		1 [ref]	1 [ref]		1 [ref]	1 [ref]	
Intermittent use	1.2 [0.9–1.6]	1.4 [0.9–2.1]		1.2 [0.9–1.5]	1.3 [0.9–2.0]		1.1 [0.9–1.5]	1.3 [0.9–1.9]		1.1 [0.8–1.5]	1.4 [0.9–2.2]	
Intro. <4 mo and perst.	1.1 [0.9–1.3]	1.5 [1.1–1.9]		1.1 [0.9–1.3]	1.4 [1.1–1.9]		1.1 [0.9–1.3]	1.4 [1.1–1.8]		1.1 [0.9–1.3]	1.5 [1.1–2.1]	
Intro. ≈5 mo and perst.	1.1 [1.0–1.2]	1.3 [1.1–1.6]		1.1 [0.9–1.2]	1.3 [1.1–1.5]		1.1 [0.9–1.2]	1.3 [1.1–1.5]		1.1 [0.9–1.2]	1.4 [1.1–1.7]	
Intro. ≈7 mo and perst.	1.1 [1.0–1.3]	1.2 [1.0–1.4]		1.1 [1.0–1.3]	1.2 [1.0–1.5]		1.1 [1.0–1.3]	1.2 [1.0–1.5]		1.2 [1.0–1.3]	1.4 [1.2–1.8]	
Complementary feeding introduction excluding baby cereals			<10^−3^			<10^−3^			<10^−3^			0.01
<4 mo	1.0 [0.8–1.3]	1.4 [1.1–1.9]		1.0 [0.8–1.3]	1.4 [1.1–1.9]		1.0 [0.8–1.2]	1.4 [1.1–1.9]		1.0 [0.8–1.2]	1.3 [0.9–1.8]	
4–6 mo	1 [ref]	1 [ref]		1 [ref]	1 [ref]		1 [ref]	1 [ref]		1 [ref]	1 [ref]	
>6 mo	0.9 [0.8–1.0]	0.8 [0.6–1.0]		0.9 [0.8–1.0]	0.8 [0.6–0.9]		0.9 [0.8–1.0]	0.8 [0.6–0.9]		0.9 [0.8–1.0]	0.7 [0.6–0.9]	
Trajectories of use of thickened formula between 2 and 10 mo			0.01			0.01			0.01			0.04
Never	1 [ref]	1 [ref]		1 [ref]	1 [ref]		1 [ref]	1 [ref]		1 [ref]	1 [ref]	
Only <6 mo	1.1 [0.9–1.4]	1.4 [1.0–1.8]		1.1 [0.9–1.3]	1.3 [1.0–1.7]		1.1 [0.9–1.3]	1.3 [1.0–1.7]		1.1 [0.9–1.3]	1.4 [1.0–1.9]	
Intro. ≈4 mo and perst.	1.1 [0.9–1.3]	0.7 [0.5–1.1]		1.0 [0.8–1.3]	0.7 [0.5–1.0]		1.0 [0.8–1.3]	0.7 [0.5–1.0]		1.1 [0.9–1.3]	0.8 [0.5–1.2]	
Intro. ≈6 mo and perst.	0.8 [0.7–1.0]	0.8 [0.6–1.0]		0.8 [0.7–1.0]	0.8 [0.6–1.0]		0.8 [0.7–0.9]	0.8 [0.6–1.0]		0.8 [0.7–1.0]	0.8 [0.6–1.1]	
Always	0.9 [0.8–1.1]	1.0 [0.8–1.3]		0.9 [0.8–1.1]	1.0 [0.8–1.3]		0.9 [0.8–1.1]	1.0 [0.8–1.3]		1.0 [0.8–1.1]	1.1 [0.8–1.4]	

*Note.* Data are adjusted odds ratios (aORs) [95% confidence intervals (CIs)]. The multinomial multiple logistic regression (Model 1) was adjusted for maternal characteristics (country of birth, BMI before pregnancy, psychological difficulties during pregnancy, smoking status during pregnancy, primiparous, age at delivery, education attainment, working status, single), familial income, and infant characteristics (sex, birth weight, and gastro‐intestinal reflux) and variables related to study design (maternal region of residence, size of maternity unit and wave of recruitment). Significant association (*p* < .05).

Abbreviations: mo, month; intro., introduction; perst., persistence.

^a^ Model 2: Model 1also adjusted on night waking at 2 months.

^b^ Model 3: Model 2 also adjusted on the use of sucking the pacifier/thumb at 1 year.

^c^ Model 4: Model 3 also adjusted on the infant sleep‐related variables (sleep arrangement, sleeping in parent's room and parental presence).

After adjustment on night waking at 2 months (Model 2) and on the use of a pacifier or sucking the thumb to fall asleep (Model 3), associations remained similar. However, the association between breastfeeding ≥6 months or early CFI (>4 months) and sleep onset difficulties were no longer significant after further adjustment on infant sleep‐related variables at age 1 (Model 4).

Breastfeeding duration ≥6 months was associated with parental presence to fall asleep at age 1 (38.5% yes vs. 21.6% no), and their interaction was significant for sleep onset difficulties (*p* < 10^−4^); the analyses were then stratified on parental presence to fall asleep at age 1 for the last model. Breastfeeding durations, whatever the parental presence, were not associated with sleep onset difficulties except for 3 to <6 months. Among infants who needed parental presence to fall asleep, breastfeeding duration between 3 and <6 months was associated with increased odds of sleep onset difficulties (OR_few_ = 2.1 [1.3–3.7] and OR_frequent_ = 1.9 [1.1–3.3]), whereas breastfeeding duration ≥6 months was not associated with odds of having sleep onset difficulties (OR_few_ = 1.0 [0.6–1.6] and OR_frequent_ = 1.2 [0.7–1.9]). Among infants who did not need parental presence to fall asleep, no association was found between 3 and <6 months breastfeeding duration and sleep onset difficulties (OR_few_ = 1.0 [0.8–1.1] and OR_frequent_ = 0.8 [0.5–1.2]). No interaction was found between breastfeeding duration and pacifier use.

Sensitivity analyses on imputed data provided similar results (Table S3).

## 4 DISCUSSION

For our cohort, characterized by a high socio‐economic level, breastfeeding duration ≥6 months was related to more frequent parent‐reported night waking, sleep onset difficulties and sleep duration of ≤12 h/24 h at age 1. However, infant sleep‐related conditions such as parental practices at sleep onset accounted for the associations with sleep duration and sleep onset difficulties. An early use of baby cereals (around 5 months or earlier) was associated with poor sleep quantity or quality, whatever the model considered. An early age at CFI excluding baby cereals (<4 months) was associated with short sleep duration (≤12 h/24 h) and more frequent sleep onset difficulties but not to night waking at age 1. Finally, an early use of thickened formula (only <6 months) was related to poor sleep quality at age 1 (night waking and sleep onset difficulties), whereas a late use of thickened formula (6 months and persistent) was associated with better sleep. The use of thickened formula was not related to parent‐reported sleep quantity.

Breastfeeding duration has been found positively associated with night waking at 6 months in cross‐sectional studies from Australia (Galbally et al., 2013) and the Asia‐Pacific region (Ramamurthy et al., 2012). In older infants, Brown and Harries (2015) showed no cross‐sectional association between current breastfeeding at age 1 and night‐waking at the same age; and longitudinal studies reported no association between breastfeeding duration and sleep duration at age 1 (Nevarez et al., 2010) or night waking and sleep duration in later childhood (i.e., between 2 and 5 years; Murcia et al., 2019; Nevarez et al., 2010). In the present study, the frequency of sleep problems (defined as sleep duration ≤12 h/24 h, night waking >2 nights/week and difficulties to fall asleep) was relatively low. However, these problems were more frequently found in breastfed infants ≥6 months old. Breastfed infants may connect nursing with falling asleep and therefore require their mother to feed them before they are able to fall asleep after night waking (DeLeon & Karraker, 2007). Also, cultural values and beliefs are embedded in how parents manage and perceive aspects regarding the infant's sleep (Milan, Snow, & Belay, 2007). These aspects could explain the infant sleep‐related conditions especially those linked with infant sleep locations and bedtime routines, as shown in our previous study (Messayke et al., 2019). The results suggest that it is not breastfeeding or duration of breastfeeding by themselves that lead to sleep problems but other parental practices related to breastfeeding and to the period around sleep onset are involved. Our results and especially the adjustment on parental practices strongly argue for this hypothesis. The stratification on parental presence to fall asleep at age 1 has shown that breastfeeding duration between 3 to <6 months was associated with increased odds of sleep onset difficulties but no longer breastfeeding duration. The link between breastfeeding and sleep onset difficulties seems explained more by maintaining the parental presence beyond weaning than being present for breastfeeding. To deal with individual sleep problems, it would be important that parents discuss with a medical practitioner.

Although international guidelines recommend introducing solid foods to healthy children not earlier than 4 or 6 months of age (Fewtrell et al., 2017; World Health Organization, 2003), parents seem to introduce solids before age 4 months in part to help children sleep better at night (Brown & Rowan, 2016; Crocetti, Dudas, & Krugman, 2004). A secondary analysis of children included in a population‐based randomized clinical trial on early food introduction, especially allergenic food, conducted in England found that infants who were introduced to solid foods before age 4 months slept 7 min more on average at night in the intention‐to‐treat analysis (Perkin et al., 2018). However, only 42% of the children achieved the protocol in the early introduction group. In older children, Murcia et al. (2019) showed that early CFI excluding baby cereals (<4 months) was related to reduced risk of belonging to the short sleep duration trajectory between age 2 and 5 years. However, different authors showed that early introduction of solid food before 4 months of age was associated with short sleep duration for children aged 1 and 2 years (Brown & Harries, 2015; Murcia et al., 2019; Nevarez et al., 2010). We here showed that the age at CFI excluding baby cereals was not associated with infant night waking frequencies at age 1 nor with sleep duration after accounting for all confounding factors. Nevertheless, we showed that late CFI (>6 months) was associated with reduced odds of sleep onset difficulties at age 1. The analysis of infant's sleep is quite different among the studies, leading to incomparable results.

We also found that the early use of baby cereals (around 5 months or earlier) was associated with poor sleep quantity or quality at age 1. In very young infants, Macknin et al. (1989) showed that feeding infants cereals in the bottle before bedtime was not associated with infant's sleep at age 5 weeks or 4 months. This lack of association was also observed in later infancy in the EDEN study, showing no association between the age of baby cereal introduction with sleep quantity or quality trajectories between age 2 and 5 years (Murcia et al., 2019). However, contrary to the previous studies that defined threshold of first use, we modelled the use of baby cereal over the time from 3 months up to 10 months and were able to identify specific trajectories associated with sleep characteristics. The difference with previous published results could be the differential categorization of the children belonging to the intermittent trajectory who would have been included in an early introduction category if we had used thresholds. Additionally, we could not exclude the totality of the reverse causality bias, because we were not able to adjust for sleep quantity and difficulties to fall asleep at sleep at 2 months.

As in the EDEN mother–child French cohort, the use of thickened formula was not associated with infant sleep duration (Murcia et al., 2019). However, we highlighted that the early use of thickened formula (only <6 months) was associated with increased odds of poor sleep quality at age 1 year and that the late use of thickened formula (introduction at 6 months and persistence) was associated with reduced odds of poor sleep quality at age 1 year even after accounting for gastrointestinal reflux at age 2 months, whereas a previous study found no association between the age of first introduction of thickened formula and 2 to 5 years sleep quality trajectories (Murcia et al., 2019). This could be explained by the different methods used for collecting the information. In the previous study, the authors focused on the primary introduction of thickened formula, whereas in the present study we focused on the use of thickened formula during the period between 2 and 10 months.

Altogether, our results suggest that parents' beliefs and resorts to settle infants' sleep may not be accurate. Some parents use earlier solid introduction and thickened formula to help their infants get a better sleep, especially for infants with a difficult temperament (Brown & Rowan, 2016). Accounting for early night waking (2 months) did not change the observed associations between breastfeeding and sleep or between early introduction of food or use of thickened formula and sleep. However, early night waking does not represent all aspects of infants' sleep at age 2 months, and we cannot exclude residual confounding. If existing, this strategy does not seem to be effective.

In this study, we analysed how feeding practices were related to infant sleep independent of parental practices related to infant sleep. The ELFE study is based on a large sample of births allowing for good statistical power. Data collection, especially feeding practices between age 2 to 10 months, was prospective, which limits the memory bias regarding infant diet. As noted earlier, the chronology of the events is very important in this field of research. The design of our feeding‐practices data collection allowed us to use a longitudinal approach to model and identify different evolutions over time of the use of baby cereals between age 3 and 10 months and the use of thickened formula between age 2 and 10 months, adding new information on timeline of feeding practices and associated sleep characteristics. The complete‐case results were consistent with the multiple imputations data (imputed results available on request).

Nonetheless, some limitations need to be recognized. Sleep was collected with parental questionnaires and was subjective, possibly leading to overestimation of sleep duration and under‐ or overestimation of night waking and sleep onset difficulties. Night‐time for night waking frequency and a long‐time period for sleep onset difficulties were not defined in the questionnaire; thus, what was considered night waking or a long‐time period for sleep onset difficulties was assessed on the basis of parents' understanding and may reflect their perception of problematic night waking or sleep onset difficulties. This perception may differ in families, but we have adjusted our models on many confounding factors, including maternal and family characteristics, to take into account these differences. Objective sleep measure through actigraphy would have provided better estimations of sleep characteristics but was not considered in this nationwide study including about 10,000 infants for cost and logistic issues. Nevertheless, the use of questionnaires for sleep characteristics estimation is a classical approach used in large epidemiology studies (Dias, Figueiredo, Rocha, & Field, 2018; Galbally et al., 2013; Galland, Taylor, Elder, & Herbison, 2012). We tried to address the reverse causation bias by adjusting on infant sleep characteristics at 2 months. However, we had only the information on night waking without a complete vision on the sleep duration or the sleep onset difficulties at this age. The change in data collection between age 2 months (phone interview) and 3 to 10 months (paper or internet questionnaire) affected the sample size in that we noted a decrease in the sample size as well as an increase in the missing data. Yet we were able to compute trajectories for the food questionnaires (the use of thickened formula and the use of baby cereals) in order to evaluate the evolution of the consumption over the time. Predominant or exclusive breastfeeding duration would be more relevant to assess the association with sleep than any breastfeeding duration. However, in France, any breastfeeding initiation is relatively low (70%) and the median duration of predominant breastfeeding is about 7 weeks (Wagner et al., 2015), so we considered only any breastfeeding duration in the present study.

## 5 CONCLUSION

This study contributed to clarify the associations between feeding practices at an early age and sleep quantity or quality at age 1 while accounting for parental practices related to infant sleep. Despite the international recommendations on feeding practices, parents seem to introduce solids or add cereals before 4 months, possibly to help the infant sleep. We show that these practices are not related to a better sleep. We also show that infants' sleep habits accounted for a large part of the relation between breastfeeding duration (≥6 months) and sleep troubles at age 1 year. Recommendations on sleep habits and difference between nursing and feeding may be needed, especially for mothers who are planning to breastfeed for a long time. Follow‐up studies are needed to determine whether the relations persist in later age.

## CONFLICTS OF INTEREST

The authors declare that they have no conflicts of interest.

## CONTRIBUTIONS

SM analysed the data and drafted the initial manuscript under the supervision of SP and BLG. CDP, SN, MND and MAC gave guidance for the interpretation of the results. MAC designed and coordinated the data collection. All authors are responsible for the reported research, have reviewed approved the final manuscript as submitted.

## Supporting information


**Figure S1.** Flow chart of selection of analysis populationTable S1. Criteria of the best model performance of trajectories
Table S2. Bivariate analysis between infant feeding practices and sleep quantity/quality parameters
Table S3. Multiple logistic regressions on imputed data for model 4 (*N* = 9,142)
