# Supplementary material for: Infant feeding practices and sleep at 1 year of age in the nationwide ELFE cohort
Source: Matern Child Nutr. 2020 Sep 10;17(1):e13072. doi: 10.1111/mcn.13072 (PMC7729538; doi:10.1111/mcn.13072)
Supplement: Supplementary file 1 — Figure S1. Flow chart of selection of analysis populationTable S1. Criteria of the best model performance of trajectories Table S2. Bivariate analysis between infant feeding practices and sleep quantity/quality parameters Table S3. Multiple logistic regressions on imputed data for model 4 (N = 9,142) [file MCN-17-e13072-s001.docx]

# SUPPORTING INFORMATION

17,794 newborns

18,329 newborns

- Multiple pregnancies (n=548)
- Premature infants <37 WA (n=756)
- Parental withdrawal of consent (n=57)
- Impossible to verify the eligibility criteria due to missing data (n=478)

16,490 infants

10,309 infants

10,207 infants

10,108 infants

- Incomplete questionnaire at 1 year (n=531)
- Missing data on at least one sleep characteristics at 1 year old (n= 242)
- >4 missing data on the use of baby cereals between 3 and 10 months (n=989)
- >4 missing data on the use of thickened formula trajectory between 2 and 10 months (n=1,091)

9,335 infants

- No data on breastfeeding duration (n=84)
- No data on complementary feeding initiation (n=109)

9,142 infants

- Missing data for potential confounders (n=446)

8,696 infants

- Missing data on the trajectories of use of thickened formula between 2 and 10 months (n=201)
- Missing data on the trajectories of use of baby cereals between 3 and 10 months (n=99)

11,298 infants

- No data for the complementary feeding questionnaire (n=5,192)

**Supplementary Figure 1. Flow chart of selection of analysis population**

**Supplementary Table 1. Criteria of the best model performance of trajectories**

|  |  |  | **BIC** | **ᴨ** | **P** | **Δ prev** | **AvePP** | **OCC** |
| --- | --- | --- | --- | --- | --- | --- | --- | --- |
| Trajectories of use of thickened formula (n=10,207) | | | -24415.1 |  |  |  |  |  |
|  | Group 1 (order = 0) | Never |  | 0.554 | 0.559 | 0.005 | 0.98 | 38 |
|  | Group 2 (order = 3) | Only before 6 months |  | 0.077 | 0.072 | -0.005 | 0.94 | 192 |
|  | Group 3 (order = 3) | Introduction around 4 months and persistence |  | 0.050 | 0.051 | 0.001 | 0.88 | 139 |
|  | Group 4 (order = 3) | Introduction around 6 months and persistence |  | 0.121 | 0.115 | -0.006 | 0.94 | 125 |
|  | Group 5 (order = 3) | Always |  | 0.198 | 0.201 | 0.003 | 0.96 | 102 |
| Trajectories of use of baby cereals (n=10,309) | | | -26237.1 |  |  |  |  |  |
|  | Group 1 (order = 3) | Never |  | 0.349 | 0.370 | 0.021 | 0.91 | 19 |
|  | Group 2 (order = 2) | Intermittent use |  | 0.041 | 0.032 | -0.009 | 0.83 | 114 |
|  | Group 3 (order = 3) | Introduction before 4 months and persistence |  | 0.111 | 0.100 | -0.011 | 0.97 | 259 |
|  | Group 4 (order = 3) | Introduction around 5 months and persistence |  | 0.213 | 0.240 | 0.027 | 0.83 | 18 |
|  | Group 5 (order = 3) | Introduction around 7 months and persistence |  | 0.284 | 0.254 | -0.030 | 0.92 | 29 |

Note. BIC: Bayesian Information Criteria, P: estimated prevalence.

The quality of the best model, regarding the number of groups and the shape of the trajectories, was verified according to recommended criteria (average posterior probability (AvePP ≥0.7), odds of correct classification (OCC ≥5), and similarity between the model's estimation of prevalence and the actual prevalence (Δ prev)).

**Supplementary Table 2. Bivariate analysis between infant feeding practices and sleep quantity/quality parameters**

|  |  | Total sleep duration-24h | | |  | Night waking | | |  | | Sleep onset difficulties | | | |  | |  |
| --- | --- | --- | --- | --- | --- | --- | --- | --- | --- | --- | --- | --- | --- | --- | --- | --- | --- |
|  |  | ≤12 h | <12 to 14 h | > 14 h | p-value^a^ | Never | 1-2 nights/wk | >2  nights/wk | | p-value^a^ | | Never | Sometimes | Always | | p-value^a^ | |
| Breastfeeding duration | |  |  |  | **10 ^-5^** |  |  |  | | **10 ^-3^** | |  |  |  | | **10 ^-3^** | |
|  | Never | 13.3 (271) | 49.8 (1,016) | 36.9 (752) |  | 50.8 (1,036) | 33.2 (677) | 16.0 (326) | |  | | 54.3 (1,108) | 36.7 (748) | 9.0 (183) | |  | |
|  | <1 mo | 15.1 (211) | 48.4 (674) | 36.5 (508) |  | 50.2 (699) | 32.7 (455) | 17.1 (239) | |  | | 49.0 (683) | 39.8 (554) | 11.2 (156) | |  | |
|  | 1 to <3 mo | 14.1 (199) | 50.1 (708) | 35.8 (506) |  | 48.2 (680) | 35.2 (498) | 16.6 (235) | |  | | 51.2 (723) | 38.0 (537) | 10.8 (153) | |  | |
|  | 3 to <6 mo | 13.2 (221) | 51.0 (857) | 35.8 (602) |  | 50.0 (840) | 31.2 (524) | 18.8 (316) | |  | | 50.5 (848) | 38.5 (648) | 11.0 (184) | |  | |
|  | ≥ 6 mo | 17.2 (374) | 52.6 (1,143) | 30.2 (654) |  | 41.9 (910) | 30.3 (657) | 27.8 (604) | |  | | 44.5 (966) | 37.0 (803) | 18.5 (402) | |  | |
| Trajectories of use of baby cereals between 3 and 10 mo | |  |  |  | **10 ^-3^** |  |  |  | | **10 ^-3^** | |  |  |  | | **0.04** | |
|  | Never | 13.1 (423) | 51.5 (1,658) | 35.4 (1141) |  | 49.4 (1,593) | 30.8 (993) | 19.8 (636) | |  | | 52.0 (1676) | 36.9 (1,188) | 11.1 (358) | |  | |
|  | Intermittent use | 16.4 (45) | 50.9 (140) | 32.7 (90) |  | 43.7 (120) | 32.7 (90) | 23.6 (65) | |  | | 47.3 (130) | 38.9 (107) | 13.8 (38) | |  | |
|  | Intro. <4 mo and persist. | 19.7 (156) | 46.4 (368) | 33.9 (269) |  | 44.3 (352) | 31.7 (251) | 24.0 (190) | |  | | 48.4 (384) | 37.2 (295) | 14.4 (114) | |  | |
|  | Intro. ≈5 mo and persist. | 15.6 (331) | 50.2 (1,062) | 34.2 (725) |  | 46.3 (980) | 34.1 (722) | 19.6 (416) | |  | | 49.2 (1,041) | 37.7 (799) | 13.1 (278) | |  | |
|  | Intro. ≈7 mo and persist. | 14.1 (321) | 51.1 (1,170) | 34.8 (797) |  | 49.0 (1,120) | 33.0 (755) | 18.0 (413) | |  | | 47.9 (1,097) | 39.4 (901) | 12.7 (290) | |  | |
| Complementary feeding introduction excluding baby cereals | |  |  |  | **10 ^-5^** |  |  |  | | **10 ^-3^** | |  |  |  | | **10 ^-4^** | |
|  | <4 mo | 22.2 (102) | 49.8 (229) | 28.0 (129) |  | 40.4 (186) | 37.4 (172) | 22.2 (102) | |  | | 44.2 (203) | 36.5 (168) | 19.3 (89) | |  | |
|  | 4-6 mo | 14.3 (970) | 50.8 (3438) | 34.9 (2,359) |  | 48.1 (3,253) | 32.5 (2,196) | 19.4 (1,318) | |  | | 49.6 (3,355) | 38.1 (2,577) | 12.3 (835) | |  | |
|  | >6 mo | 13.9 (204) | 49.8 (731) | 36.3 (534) |  | 49.4 (726) | 30.2 (443) | 20.4 (300) | |  | | 52.4 (770) | 37.1 (545) | 10.5 (154) | |  | |
| Trajectories of use of thickened formula between 2 and 10 mo | |  |  |  | 0.11 |  |  |  | | **10 ^-6^** | |  |  |  | | **10 ^-4^** | |
|  | Never | 14.7 (717) | 50.4 (2,448) | 34.9 (1,700) |  | 47.5 (2,313) | 31.1 (1,509) | 21.4 (1,043) | |  | | 48.6 (2,366) | 38.2 (1,855) | 13.2 (644) | |  | |
|  | Only <6 mo | 17.6 (113) | 51.3 (329) | 31.1 (199) |  | 42.6 (273) | 38.5 (247) | 18.9 (121) | |  | | 45.7 (293) | 39.6 (254) | 14.7 (94) | |  | |
|  | Intro. ≈4 mo and persist. | 13.9 (63) | 52.5 (238) | 33.6 (152) |  | 48.3 (219) | 31.2 (141) | 20.5 (93) | |  | | 49.9 (226) | 41.7 (189) | 8.4 (38) | |  | |
|  | Intro. ≈6 mo and persist. | 13.5 (135) | 53.1 (532) | 33.4 (334) |  | 49.3 (493) | 35.1 (352) | 15.6 (156) | |  | | 54.4 (545) | 34.9 (349) | 10.7 (107) | |  | |
|  | Always | 14.3 (248) | 49.0 (851) | 36.7 (637) |  | 49.9 (867) | 32.4 (562) | 17.7 (307) | |  | | 51.7 (898) | 37.1 (643) | 11.2 (195) | |  | |

Values are % (n). Note: ^a^Based on chi-square test; mo: month, intro.: introduction, perst.: persistence. In bold: significant modality (p<.05)

**Supplementary Table 3. Multiple logistic regressions on imputed data for model 4 (N=9,142)**

|  |  | Total sleep duration^a^ | | Night waking^b^ | | Sleep onset difficulties^b^ | |
| --- | --- | --- | --- | --- | --- | --- | --- |
|  |  | ≤12 h | > 14 h | 1-2 nights/week | >2 nights/week | Sometimes | Always |
| Breastfeeding duration | |  |  |  |  |  |  |
|  | Never | 1.0 [0.8–1.2] | 0.9 [0.8–1.1] | 0.9 [0.8–1.1] | 1.0 [0.8–1.2] | 0.9 [0.8–1.1] | 0.8 [0.6–1.1] |
|  | <1 mo | 1.1 [0.9–1.4] | 1.0 [0.9–1.2] | 0.8 [0.7–1.0] | 0.9 [0.7–1.2] | 1.1 [0.9–1.3] | 1.0 [0.7–1.3] |
|  | 1 to <3 mo | 1 [Ref] | 1 [Ref] | 1 [Ref] | 1 [Ref] | 1 [Ref] | 1 [Ref] |
|  | 3 to <6 mo | 0.9 [0.7–1.1] | 1.1 [0.9–1.2] | 0.9 [0.8–1.1] | 1.0 [0.8–1.3] | 1.0 [0.9–1.2] | 1.0 [0.8–1.3] |
|  | ≥ 6 mo | 0.9 [0.7–1.1] | 1.0 [0.8–1.1] | 1.1 [0.9–1.3] | **1.4 [1.2–1.8]** | 1.0 [0.9–1.2] | 1.2 [0.9–1.6] |
| Trajectories of use of baby cereals between 3-10 mo | |  |  |  |  |  |  |
|  | Never | 1 [Ref] | 1 [Ref] | 1 [Ref] | 1 [Ref] | 1 [Ref] | 1 [Ref] |
|  | Intermittent use | 1.1 [0.8–1.6] | 0.9 [0.7–1.2] | 1.1 [0.8–1.4] | 1.4 [1.0–1.9] | 1.1 [0.9–1.5] | 1.3 [0.8–2.1] |
|  | Intro. <4 mo and perst. | **1.5 [1.2–1.9]** | 1.0 [0.8–1.1] | 1.0 [0.9–1.3] | **1.5 [1.2–1.9]** | 1.0 [0.9–1.3] | **1.5 [1.1–2.0]** |
|  | Intro. ≈5 mo and perst. | 1.2 [0.9–1.4] | 0.9 [0.8–1.1] | 1.1 [1.0–1.3] | 1.1 [1.0–1.3] | 1.0 [0.9–1.2] | **1.4 [1.2–1.8]** |
|  | Intro. ≈7 mo and perst. | 1.1 [0.9–1.3] | 1.0 [0.8–1.1] | 1.1 [0.9–1.2] | 1.0 [0.8–1.1] | **1.2 [1.0–1.3]** | **1.5 [1.2–1.8]** |
| Complementary feeding intro. excluding baby cereals | |  |  |  |  |  |  |
|  | <4 mo | 1.2 [0.9–1.5] | 0.8 [0.6–1.0] | 1.2 [0.9–1.5] | 1.0 [0.7–1.3] | 1.0 [0.8–1.2] | 1.3 [0.9–1.8] |
|  | 4-6 mo | 1 [Ref] | 1 [Ref] | 1 [Ref] | 1 [Ref] | 1 [Ref] | 1 [Ref] |
|  | >6 mo | 1.1 [0.9–1.3] | 1.1 [1.0–1.3] | 0.9 [0.8–1.1] | 1.0 [0.8–1.16] | 0.9 [0.8–1.0] | **0.7 [0.6–0.9]** |
| Trajectories of use of thickened formula between 2 and 10 mo | |  |  |  |  |  |  |
|  | Never | 1 [Ref] | 1 [Ref] | 1 [Ref] | 1 [Ref] | 1 [Ref] | 1 [Ref] |
|  | Only <6 mo | 1.2 [0.9–1.5] | **0.8 [0.6–0.9]** | **1.3 [1.1–1.6]** | 1.0 [0.8–1.3] | 1.1 [0.9–1.3] | 1.3 [1.0–1.8] |
|  | Intro. ≈4 mo and perst. | 1.1 [0.8–1.5] | 0.9 [0.7–1.1] | 1.0 [0.8–1.3] | 1.2 [0.9–1.6] | 1.0 [0.8–1.3] | 0.7 [0.5–1.1] |
|  | Intro. ≈6 mo and perst. | 1.0 [0.8–1.2] | 0.9 [0.8–1.0] | 1.1 [0.9–1.3] | **0.8 [0.6–0.9]** | **0.8 [0.7–0.9]** | 0.8 [0.6–1.1] |
|  | Always | 1.0 [0.9–1.3] | 0.9 [0.8–1.1] | 1.0 [0.8–1.1] | 0.9 [0.8–1.1] | 0.9 [0.8–1.1] | 1.1 [0.8–1.4] |

Data are adjusted odds ratios (aORs) [95% confidence intervals (CIs)]

Note. aOR, adjusted odds ratio; CI 95%, confidence interval 95%, mo: month, intro.: introduction, perst.: persistence; in bold: significant modality (p< .05)

^a^reference: <12 to 14 h; ^b^reference: never

Adjusted on: maternal characteristics, familial income, infant characteristics, night waking at 2 months, use of sucking the pacifier/thumb at 1 year, and infant sleep-related variables and variables related to study design.
